# Stability Analysis and Multi-Trait Selection of Flowering Phenology Parameters in Olive Cultivars Under Multi-Environment Trials

**DOI:** 10.3390/plants14131906

**Published:** 2025-06-20

**Authors:** Jinhua Li, Dongxu Jia, Zhenyuan Zhou, Jincheng Du, Qiangang Xiao, Mingrong Cao

**Affiliations:** 1 State Key Laboratory of Tree Genetics and Breeding, Research Institute of Forestry, Chinese Academy of Forestry, Beijing 100091, China; 19831863762@163.com (D.J.); shimlyzzy@163.com (Z.Z.); cmr0511@126.com (M.C.); 2 Key Laboratory of Tree Breeding and Cultivation, State Forestry and Grassland Administration, Beijing 100091, China; 3 Sichuan Academy of Forestry Sciences, Chengdu 610081, China; 18190770069@163.com; 4 Forestry Research Institute, Chengdu Academy of Agricultural and Forestry Sciences, Chengdu 611130, China; xiaoqg1992@163.com

**Keywords:** olive tree flowering phenology, multi-environment trial (MET), GEI, GGE-biplot, weighted average of absolute scores (WAASB), MPS, MTMPS

## Abstract

Flowering represents the most important process in the reproductive stage of fruit trees, including olive trees. Previous studies have demonstrated that the genotype–environment interaction (GEI) has a considerable influence on olive flowering time. This study investigated the GEI and genetic parameters influencing olive flowering phenology in Southwestern China (a non-Mediterranean region), using multi-trait-based stability selection methods. Sixteen olive cultivars from five countries were evaluated over two years in two distinct climatic regions of Southwestern China. Flowering phenology was assessed based on three parameters: full-bloom date (FBD), flowering-period length (FP), and full-bloom-period length (FBP). In the analyses, the best linear unbiased prediction (BLUP) to predict genetic value and genotype + genotype by environment interaction (GGE) biplot methods to visualize and assess stability and performance were employed across four environments. The results showed that genotype, environment, and GEI had highly significant effects on flowering traits, with GEI accounting for 54.12% to 89.62% of the variance. Heritability values were low (0.0589 to 0.262), indicating that genetic factors had limited control over flowering phenology compared to environmental factors. A stability analysis using a mean performance and stability (MPS) index identified genotypes with earlier flowering dates and longer flowering periods. Multi-trait selection using a multi-trait mean performance and stability (MTMPS) index further highlighted six superior genotypes with high performance and stability across environments. The findings emphasize the critical role of environmental factors on olive flowering phenology, highlighting the challenges in breeding for stable flowering traits. This study demonstrates the effectiveness of multi-trait selection methods in identifying genotypes with superior performance and stability under different environmental conditions. These results provide valuable insights for olive breeding programs, particularly in non-Mediterranean regions, suggesting that targeted selection and multi-trait evaluation could enhance the adaptability and productivity of olive cultivars under changing climatic conditions.

## 1. Introduction

Olive (*Olea europaea* L.), the most characteristic fruit and oil crop in the Mediterranean Basin (MB), is well adapted to the climate [1,2]. Over the past few decades, olive oil’s global popularity has expanded its cultivation beyond the MB to regions and countries with different temperature and precipitation patterns [2,3,4,5,6]. While olive trees have the capacity to adapt to diverse environments due to their unique traits [7,8], climate change highlights the need to understand the genetic and environmental factors affecting production [5,9,10,11]. Temperature drives olive tree phenology, which varies by variety and is influenced by environmental factors [11,12,13,14]. Understanding olive trees’ reproductive biological responses in new growing regions is crucial for sustainable oil productivity and quality [2,7,15,16].

Flowering is a critical reproductive process for many fruit trees, including olives, as most cultivars are self-sterile and require cross-pollination [1,17]. Studies have shown significant variability in flowering phenology among olive cultivars, particularly in flowering timing [5,12,18,19,20,21]. Optimal flowering timing is essential to avoid adverse weather conditions that impact flower quality and fruit set, influencing olive cultivation, variety improvement, and breeding [16,22,23,24,25,26]. Genetic variability is key in breeding programs to enhance yield [23] and differences in flowering phenology can be used to select cultivars with ideal flowering times [5,23,27,28]. Flowering time, influenced by both genetic and environmental factors, is crucial for olive tree adaptation and productivity. Extensive research has focused on the genetic factors influencing flowering time, with numerous studies reporting the significant impact of specific genes in this process [29,30,31].

Various studies have made significant contributions to this area, such as those conducted by Garcia-Mozo et al. [19], De Melo-Abreu et al. [20], Mancuso et al. [32], and Alcalá and Barranco [21]. Furthermore, environmental factors have also been demonstrated to exert a significant influence [5,23,33,34,35,36,37]. Previous studies have demonstrated that the GEI exerted a considerable influence on olive flowering time [27]. However, the variability in olive cultivars under identical environmental conditions had been the subject of only a limited number of studies [19,22,23,38]. Thus, this subject has received only a modicum of attention to date, although studies have indicated the influence of this factor on olive trees. Furthermore, there is still a paucity of information on the effect of the GEI on flowering time [5,22,27,30].

Most variability in flowering phenology is due to environmental factors [27,38]. Multi-environment trials (METs) help compare the impacts of genetic and environmental factors and their interaction (GEI) on agronomic traits [39,40]. GEI, defined as genotype performance variation due to different environments, is critical for cultivar performance [41,42,43] and stability in METs [39,44,45,46], and affects selection efficacy in breeding programs [40,42,47]. Identifying GEI in specific or broad environments is vital for accurately evaluating genotype performance, including that of olives [27,38,42]. Assessing genotype performance across multiple locations or years provides insights into olive cultivar adaptation and stability [5,7,12,22,23,27,38]. While studies have evaluated olive cultivars in the MB [5,14,27,38,48], a few have used data from non-Mediterranean environments [2,3,4,5,12,34,36,49]. There is limited information on GEI extent and pattern, as well as genotype stability across diverse regions, especially for genotypes with consistent performance in varied conditions.

Genotype stability across locations or varying environmental conditions can be assessed through G×E modeling in METs [40,42,43]. Over the past few decades, numerous statistical models and approaches have been developed for GEI analysis [40] and for identifying high-yielding, stable genotypes to aid breeding programs in cultivar selection and environment recommendations [39,41,44,45,50]. GEI can be evaluated through numerical and graphical methods to identify high-performing, stable genotypes across varied environments. The GGE biplot method is frequently applied to assess GEI in crops, either independently or integrated with other approaches, and has proven effective for analyzing target trait performance in METs [41,42,45,51]. In addition to exhibiting agronomic potential in specific target environments, olive cultivars must also demonstrate phenotypic stability in order to be suitable for and adapt to a range of geographical areas. A simultaneous evaluation of multiple desirable traits would be an efficient approach to genotype selection. However, genotype selection with the aim of achieving high performance in multiple traits simultaneously is a complex challenge due to unwanted correlations resulting from the traits’ complex relationships and genetic architectures [41,46,52]. Few reports have examined the association between the GEI and mean performance, adaptability, and stability of genotypes for multi-trait selection in olive trials.

Olivoto et al. [46,52,53] developed two methods to incorporate weighting mean performance and stability of genotypes. The first, mean performance and stability (MPS), calculates the weighted average of absolute scores (WAASB) from singular value decomposition (SVD) of a BLUP matrix generated by a linear mixed model (LMM) for GEI [39]. WAASB has been used to identify high-yielding and stable barley genotypes [45,50] and select stable cassava genotypes under drought conditions [54]. The WAASB model effectively identified high-yielding, stable soybean genotypes in METs, demonstrating strong grouping capability by performance and stability [55]. The second method, the MTMPS index, improves upon the multi-trait stability index (MTSI), using the Euclidean distance between genotypes and ideotypes with scores derived from an exploratory factor analysis. Genotypes with the lowest MTMPS value are the closest to ideotypes, indicating superior multi-trait performance and stability [39,41,46,55,56]. The genotype with the lowest MTMPS value is closest to the ideotype, indicating high MPS across traits. Multi-trait selection using MPS and MTMPS indices has proven effective for estimating genetic parameters, capturing GEI nuances, and identifying stable, high-performing genotypes in crops like durum wheat [44], cassava breeding initiatives [39], and prominent hybrids of maize [41].

In recent decades, valuable olive cultivars have been introduced into China, with large-scale domestication beginning in 1964 through the establishment of 12 provincial plantations for regional trials [57,58]. Over 150 cultivars from Mediterranean countries were planted in northern subtropical regions with a non-Mediterranean climate. Today, the planted area exceeds 100,000 hectares, primarily in Southwestern China, across eight provinces: Gansu, Sichuan, Yunnan, Chongqing, Hubei, Hunan, Guizhou, and Shaanxi [59,60]. These regions have a subtropical climate with summer rainfall, contrasting with the winter rainfall of the MB. New climatic factors significantly impact olive tree performance, especially in flowering, fruit growth, oil production, and quality. However, many olive trees show low fruit set and unstable yields during the fruiting period. Research on olive pollination biology under multi-environmental conditions in China is limited. Thus, it is crucial to enhance fruit yield by selecting superior genotypes for the southwestern region, a major production area in China. Studying olive flowering phenology is essential for improving fruit set, genetic resource selection, and crossbreeding [23,25].

The aim of the present study was to determine the flowering phenology parameters of sixteen olive cultivars over two years in two distinct climatic regions: Longnan, Gansu Province, and Xichang, Sichuan Province, located in Southwestern China. A MET experiment was conducted by cultivating the same genotypes in disparate locations. This methodological approach provided a unique opportunity to study the effect of both genetic and environmental factors on olive flowering time. Genetic variability was represented by the 16 olive genotypes, while environmental variability was attributed to the different conditions offered by the different locations and the two different study years. The four objectives of this study were as follows: (1) to examine the GEI effect on the flowering phenology of olive genotypes; (2) to estimate the genetic parameters of flowering traits; (3) to identify groups of genotypes with high performance, adaptability, and stability using the MPS index; and (4) to select genotypes demonstrating high performance and stability in multiple traits using the MTMPS index. Additionally, the observations made in this study offer valuable insights into the characteristics of the tested olive genotypes and locations.

## 2. Results

### 2.1. ANOVA and BLUP-Based Mean Performance of Flowering Traits Across Four Environments

A joint analysis of variance (ANOVA) of the flowering parameters of sixteen olive genotypes in four distinct environments was performed, as presented in Table 1. The results showed that the effects of genotype (GEN), environment (ENV), and genotype–environment interaction (GEI) were highly significant (*p* < 0.001). These results indicate that the differences in the environments had a significant effect on the flowering parameters, followed by the GEI. The results herein can be employed to estimate the G × E interaction, stability, and genotypic response parameters in different environments.

**Figure 1:**
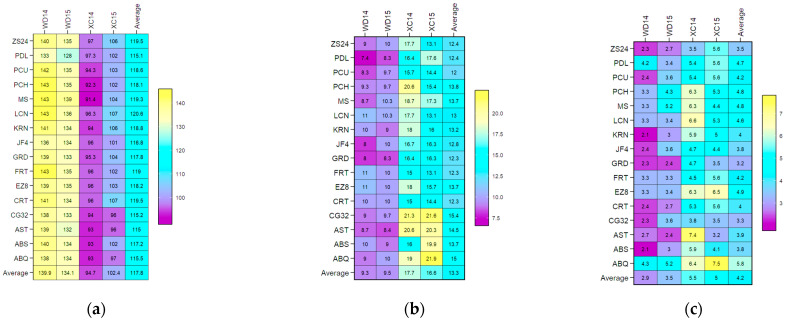
BLUP plotting the mean performance of flowering parameters in four environments for 16 olive genotypes. Abbreviations are as described in the Section 4. Note: (**a**) FBD (full-bloom time by Julian date); (**b**) FP (length of flowering period in days); (**c**) FBP (length of flowering-bloom period in days).

A heat map of the sixteen olive genotypes was constructed using BLUP data obtained from LMM in order to allow for a graphical interpretation of the genotype vs. environment interaction. This was conducted for the three flowering parameters across two growing environments at two different locations, and the resulting map is presented in Figure 1 with regard to the FP, FBP, and FBD traits. The map shows the mean performance of each olive genotype in each test environment, the overall average performance of each test genotype across all environments, and the mean performance of each test environment. The map demonstrates that the overall genotype performance varied across the test environments, thereby verifying the GEI.

The minimum BLUP value of FBD was observed in XC14 (Figure 1a) for the olive cultivar MS (91.4), while the maximum BLUP values of FP (Figure 1b) and FBP (Figure 1c) were detected for ABQ (21.9 and 7.5) in XC15. Furthermore, the lowest mean performance for the BLUP of FBD (Figure 1a) was observed in XC14 (97), while the highest mean performance for the BLUP of FP (Figure 1b) and FBP (Figure 1c) was detected in XC14 (17.7) and XC15 (5.5), respectively. Among the genotypes studied across the test environments, AST with FBD  =  115 (Figure 1a) exhibited the lowest mean BLUP (115), while CG32 with FP  =  15.4 (Figure 1b) and ABQ with FBP  =  5.8 (Figure 1c) exhibited the highest average BLUP.

### 2.2. BLUP-Based Variance Components and Genetic Parameters

**Figure 2:**
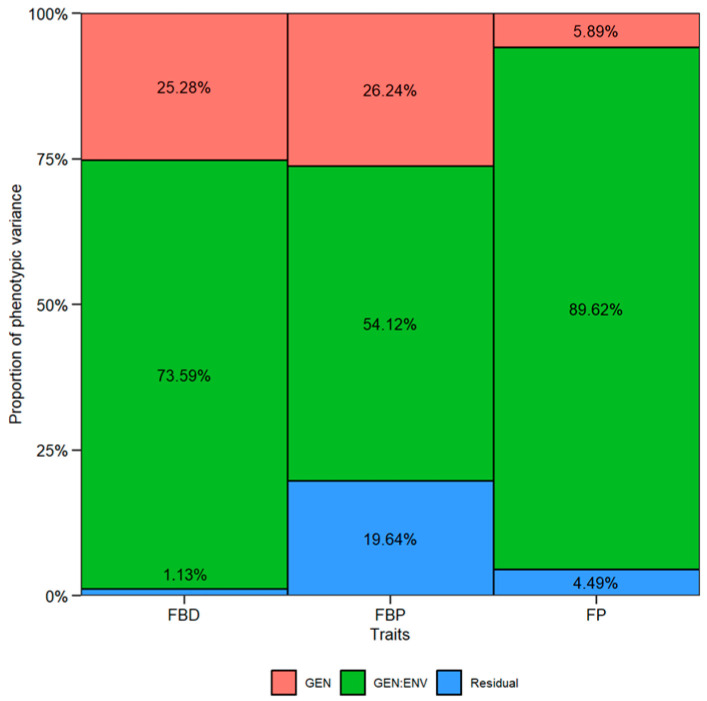
Proportion of phenotypic variance for three flowering phenology traits, FBD, FBP, and FP, evaluated across four environments. Note: FBD, full-bloom time by Julian date; FBP, length of flowering-bloom period; FP, length of full-bloom period; GEN, genotypic variance; GEN:ENV, genotype–environment interaction; Residual, environmental variance.

Figure 2 illustrates the variance components estimated by the BLUP for the flowering parameters of the olive genotypes across the four environments. It was observed that the proportion of GEI variances ranged from 73.59% to 89.62%, which was higher than that of the genotypic variances, which ranged from 5.89% to 26.24%, for all three study traits. The highest degree of genetic variance was observed in FBP (26.24%), followed by FBD (25.28%) and FP (5.89%). However, the proportion of GEI variances exhibited by FP (89.62%) was found to be the most significant when compared to that exhibited by FBD (73.59%) and FBP (54.12%).

Table 2 presents the genetic parameters of the olive flowering phenology traits. The total phenotypic variance (*σ_p_*^2^) ranged from 1.24 (FBP) to 7.08 (FBD), while the heritability ranged from 0.0589 (FP) to 0.262 (FBP). For all traits (FBD, FBP, and FP), the heritability was less than 0.3, implying that the genotypic component accounted for less than 30% of the genotype mean variance (Figure 2) and that the majority of the genotype mean variance was attributable to the GEI. The coefficient of determination for the GEI effect (GEIr^2^) was higher for FP (0.896) and FBD (0.736), indicating that the GEI played a major role in the phenotypic component. The traits exhibited high heritability on genetic mean bases (*h*^2^*_mg_* > 0.5) for FBD and FBP. The accuracy of the genotype selection (AS) of the three traits ranged from 0.453 (FP) to 0.796 (FBP), with the interaction terms for these traits exhibiting a significant contribution, thereby underscoring the environmental nature of the phenotype–genotype relationship for these traits. This finding suggests that the selection of widely adapted genotypes may pose a challenge and that targeted selection within delineated mega-environments could yield favorable outcomes. The genotypic coefficient of variation (*CVg*) showed a broad spectrum ranging from 1.14% (FBD) to 13.5% (FBP), while the residual coefficient of variation (*CVr*) demonstrated a comparable range from 0.241% (FBD) to 11.7% (FBP). Moreover, the ratio between the genotypic and residual coefficients of variation (*CV ratio*) exhibited values greater than 1 for all traits. Finally, *rge* (the magnitude of the correlation of the GEI interaction effects) was high, ranging from 0.734 to 0.985.

### 2.3. BLUP-Based GGE Biplot Analysis

**Figure 3:**
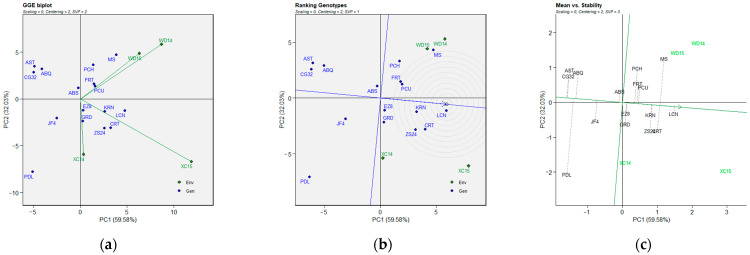
BLUP-based GGE biplot for FBD (full-bloom time by Julian date) of 16 olive genotypes across four environments. Note: (**a**) GGE biplot; (**b**) ranking of genotypes with the ideal genotype; (**c**) mean performance vs. stability. Abbreviations are as described in Section 4.

**Figure 4:**
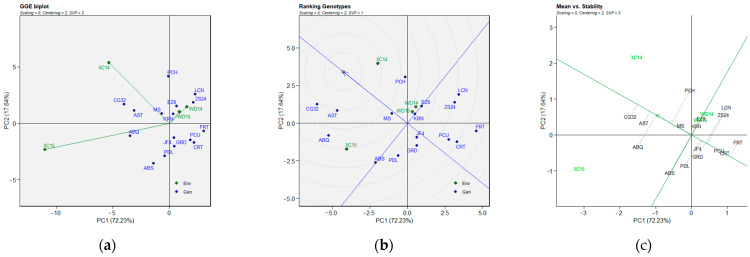
BLUP-based GGE biplot for FP (length of flowering period) of 16 olive genotypes across four environments. Note: (**a**) GGE biplot; (**b**) ranking of genotypes with the ideal genotype; (**c**) mean performance vs. stability. Abbreviations are as described in the Section 4.

**Figure 5:**
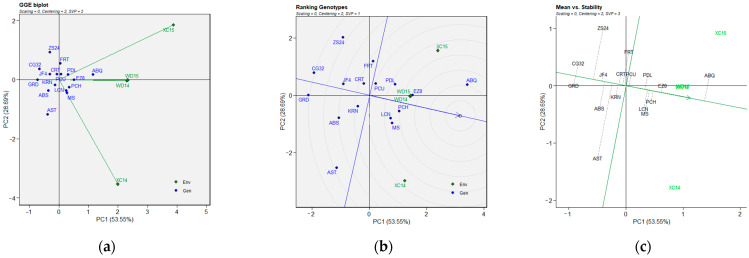
BLUP-based GGE biplot for FBP (length of full-bloom period) of 16 olive genotypes across four environments. Note: (**a**) GGE biplot; (**b**) ranking of genotypes with the ideal genotype; (**c**) mean performance vs. stability. Abbreviations are as described in Section 4.

The significant GEI effect obtained for the three parameters was assessed using a GGE biplot model based on BLUP data obtained from the LMM (Figure 3, Figure 4 and Figure 5). The biplot presents two principal components (PC1 and PC2), which explain the effects of G + GE based on their additive percentage. The biplots explained 91.61% (FBD), 89.87% (FP), and 82.24% (FBP) of the total variation observed, of which 59.58% (FBD), 72.23% (FP), and 53.55% (FBP) were explained by PC1 (axis 1), while PC2 (axis 2) explained 32.03% (FBD, Figure 3), 17.64 % (FP, Figure 4), and 28.69% (FBP, Figure 5). The line with a single arrow in the FBD (Figure 3c), FP (Figure 4c), and FBP (Figure 5c) biplots indicates the average environmental average (AEA), and it suggests an elevated mean performance across the tested genotypes. The AEAs of the two principal components (PCs) are indicated by the arrow heads in Figure 3c, Figure 4c and Figure 5c, and they are further delineated in Figure 3b, Figure 4b and Figure 5b, where the genotypes located within the circles were identified as the optimal genotypes. The line perpendicular to the AEA is designated as the average ordinate environment (AOE), while the intersection is the point that represents both the average mean performance and high stability. The other perpendicular lines connecting the genotypes to the AEA explain the stability of the genotype, with the proximity of the genotypes to the AEA indicates their stability across the environments. Regarding the “genotype ranking” GGE biplot of FBD (Figure 3b), the LCN genotype overlapped the position of the ideal genotype, while the AST and CG32 and the ABQ and EZ8 genotypes were plotted closer to the ideal genotype than the other genotypes in the GGE biplots of FP (Figure 4b) and FBP (Figure 5b), respectively. The “mean vs. stability” GGE biplot model of FP revealed that the KRN genotype exhibited a medium–high level of stability for all three parameters. The AST, PDL, and PCH genotypes demonstrated the poorest stability (i.e., high variability across environments) for FBD (Figure 3c), FP (Figure 4c), and FBP (Figure 5c), respectively. Once again, stability showed no correlation with any of the values for the flowering parameters evaluated.

### 2.4. Selection of Olive Genotypes Based on MPS Index

**Figure 6:**
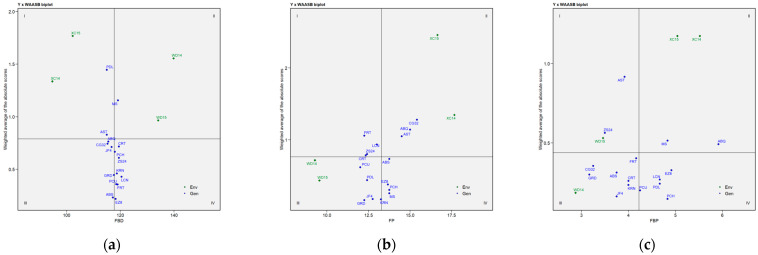
Mean performance vs. WASSB biplot based on BLUP of flowering parameters (trait) and stability. The x-axis shows the arithmetic mean of BLUP for each GEI. The y-axis shows the WAAS index. Note: WAASB, weighted average of absolute scores from the singular value decomposition (SVD) of the BLUP matrix for the GEI effects generated by the LMM. (**a**) FBD (full-bloom time by Julian date); (**b**) FP (length of flowering period in days); (**c**) FBP (length of flowering bloom period in days). Abbreviations are as described in Section 4.

**Figure 7:**
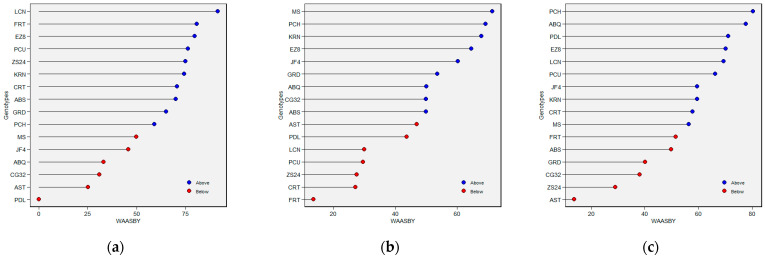
Estimated values of WAASB index for (**a**) FBD (full-bloom time by Julian date), (**b**) FP (length of flowering period), and (**c**) FBP (length of flowering-bloom period) in olive genotypes considering equal weights for mean performance and stability.

**Figure 8:**
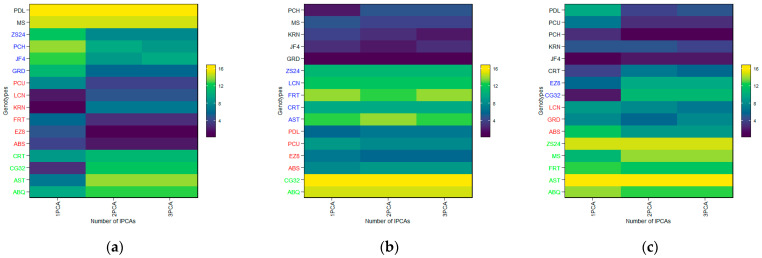
Heatmap showing the ranks of genotypes concerning the number of IPCAs used in the WAAS for the BLUPs of the genotype vs. environment interaction (WAASB) estimation. (**a**) FBD (full-bloom time by Julian date), (**b**) FP (length of flowering period), and (**c**) FBP (length of flowering-bloom period). The relative ranking of the genotype is indicated by color (intensity or hue), with darker and lighter colors reflecting higher and lower rankings, respectively.

**Figure 9:**
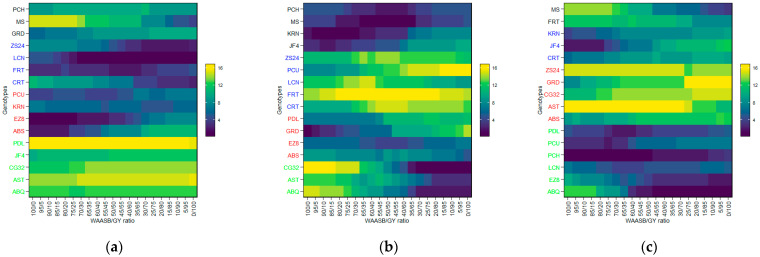
Ranks of olive genotypes considering the mean performance and stability (MPS) index with different weights for (**a**) FBD (full-bloom time by Julian date), (**b**) FP (length of flowering period), and (**c**) FBP (length of flowering-bloom period). The relative ranking of the genotype is indicated by color (intensity or hue), with darker and lighter colors reflecting higher and lower rankings, respectively.

WAASB analyses based on the MPS were utilized for the selection of olive genotypes to obtain a better and more comprehensive characterization (genotypes/environment) of the flowering phenology parameters (Figure 6, Figure 7, Figure 8 and Figure 9). The genotypes in Sector I exhibited instability, with ENVs playing the largest role in the GEI, characterized by a high discriminatory capacity (GENs and ENVs demonstrated variation according to traits). The genotypes in Sector II were unstable and highly productive, where the environment played a substantial role in the GEI, and, furthermore, the genotypes and environments varied by traits. The genotypes in Sector III were found to have a lower performance than the average due to a reduction in the WAASB values, indicating a more stable performance of genotypes across the environments (the genotypes varied by traits). The genotypes in Sector IV featured the highest and most stable performance. Consequently, the CG32, JF4, and ABQ genotypes, which exhibited the lowest BLUP values for FBD, were selected for the FBD trait (Figure 6a); PCH, MS, EZ8, and ABS were selected because they had the highest BLUP values for the FP trait (Figure 6b); EZ8, LCN, PDL, and PCU were selected because they had the highest BLUP values for the FBP trait (Figure 6c); and EZ8 was selected for each of the three flowering traits (Figure 6). The ranking of genotypes (WAASBY) is shown in Figure 7 based on the WAASB and mean performance (Y), considering equal weights for FBD (Figure 7a), FP (Figure 7b), and FBP (Figure 7c), using IPCA1.

The genotype ranking by stability is displayed in the form of a heatmap based on the number of IPCAs utilized in the WAASB assessment (see Figure 8). The relative ranking of the genotype is indicated by color (intensity or hue), with darker and lighter colors reflecting higher and lower rankings, respectively. The IPCAs employed in the WAASB assessment were applied accordingly to adjust the genotype ranking, with three IPCAs covering all traits and components. Distinction between the genotype groups was facilitated by the application of different colors, according to those exhibiting equivalent levels of performance and stability (see Figure 8). The genotypes EZ8, ABS, FRT, PCU, LCN, and GRD in Figure 8a; GRD, KRN, JF4, MS, and EZ8 in Figure 8b; and JF4, PCH, PCU, PDL, CRT, and KRN in Figure 8c were more stable than the others and obtained the lowest WAASB values within the same cluster for FBD (Figure 8a), FP (Figure 8b), and FBP (Figure 8c), respectively, (based on two or more IPCAs). With the use of IPCA1, the most visible change was observed in KRN for FBD (Figure 8a), in PCH for BP (Figure 8b), and in CG32 for FBP (Figure 8c). Alternatively, when two or more IPCAs were used, these genotypes were considered to be the most stable for these two traits, while the ability of the WAASB index to capture the variation in the IPCAs that promoted stability was reflected. The WAASB ratio of the clusters was applied to distinguish the groups of genotypes with equivalent performance; then, the optimal groups were determined to be as follows: the LCN, EZ8, and FRT genotypes for FBD (Figure 8a); the GRD, KRN, and JF4 genotypes for FP (Figure 8b), and the PCH, PDL, and PCU genotypes for FBP (Figure 8c). These outcomes were consistent or largely converged with the WAASB results (Figure 9).

### 2.5. Multi-Trait Selection for Performance and Stability Based on MTMPS Index

**Figure 10:**
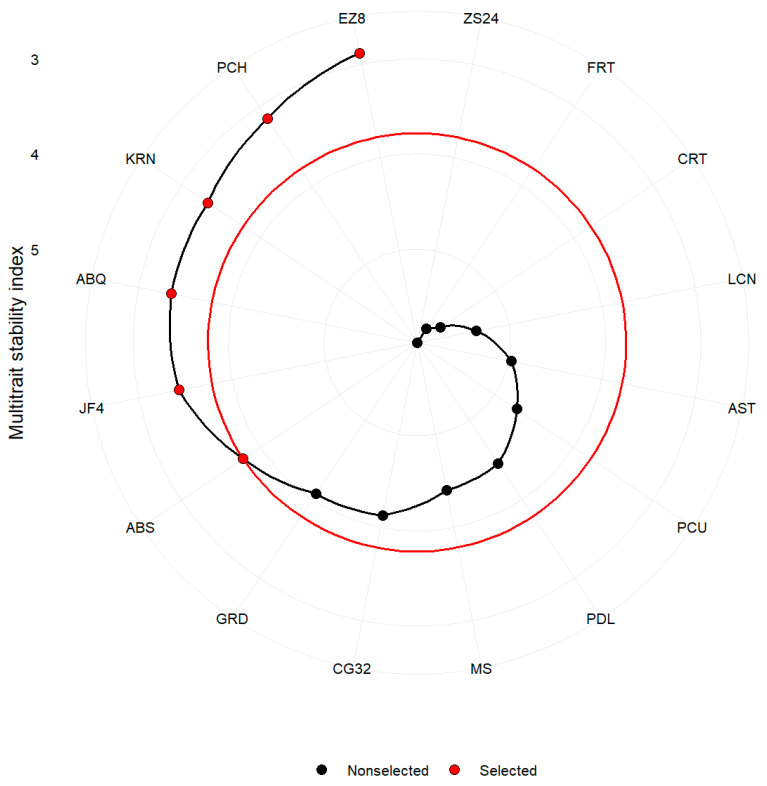
Genotype ranking and selected genotypes. The selected stable genotypes located on the red circle or beyond, with red dots, considering SI 35%.

A multi-trait stability analysis was conducted on the olive genotypes with the objective of identifying those exhibiting a long flowering-bloom period and early flowering. The sixteen olive genotypes were then ranked based on the MTMPS analysis, assuming a 35% selection intensity (SI). The results of this analysis are illustrated in Figure 10. Among the sixteen genotypes evaluated, the following six with lower MTMPS values were identified as the most stable in terms of the mean performance of multiple desired traits: EZ8 (2.883), PCH (3.151), KRN (3.335), ABQ (3.350), JF4 (3.429), and ABS (3.784). The ABS genotype was identified at the optimal cut point of the red circle in Figure 10, while the GRD genotype was closer to this red circle, suggesting that it may demonstrate stable performance in the desired traits, alongside the top six genotypes mentioned above. Consequently, in future studies, it may be interesting to examine the performance of the genotypes that were in close proximity to the cut point, taking into account the SI.

For the mean performance, the SD (selection differential) was found to be positive for the traits (flowering and blooming period) that were targeted for increase, while it was negative for the trait (flowering date) that was targeted for decrease. Higher SD values were found for FBP (0.304) and FP (0.413), which were in the common factor group FA1. The SD value of FBD was found to be −0.299, which was in the factor group FA2 (Table 3). The SD values for the mean performance were as follows: 0.413 for FP, 0.304 for FBP, and −0.299 for FBD. The percent selection differentials for the mean performance were 3.11%, 7.19%, and −0.25% for the FP, FBP and FBD traits (Table 3), respectively. The SD values for the WAASBY index were obtained for FP (13.1) and FBP (10.5), which were in the common factor group FA1 (Table 3). The SD value of FBD (7.8) was 11.5%, which was in the FA2 group (Table 3). The percentage selection differentials (SD %) values for the WAASBY index were as follows: 27.8% for FP, 18.9% for FBP, and 13.4% for FBD (Table 3).

## 3. Discussion

### 3.1. Effects of GEI and Genetic Parameters on Olive Flowering Phenology

Previous studies have evaluated the effects of genotypes [3,5,12,21,32,61,62] and the environment [3,5,12,33,48,49] on olive flowering phenology, including FBD, FP, and FBP [19]. In this study, a cultivar MET experiment compared the relative effects of genotype vs. environment and their interaction on these parameters. Results showed that the interaction of year and location significantly influenced the flowering phenology patterns, with environmental factors (geographical and climatic conditions) having a greater impact than genotype on the parameters, i.e., FBD, FP, and FBP. This confirms that flowering phenology varies significantly among different olive cultivars across years or locations [3,5,12,16,22,23,48,63,64].

The relatively lower influence of genetic factors compared to environmental factors on olive flowering phenology highlights the challenge of breeding for early-flowering cultivars. Early flowering may be desirable to avoid high temperatures and water stress, especially in a warming climate [12,23,24,38]. Identifying consistent genetic variability for this trait requires evaluating more cultivars [12,27]. A MET was used to quality the effects of GEI on flowering phenology, revealing that environmental factors have a greater impact than genetic factors, as demonstrated by Navas-Lopez et al. (2019) [27]. This suggests that breeding for early flowering may be difficult, as it is challenging to mitigate adverse effects of elevated temperatures during flowering under climate change. The significant GEI and lack of stability across flowering parameters emphasize the need for local olive trials to identify suitable cultivars for specific environments [22,23].

The evaluation of different genotypes in this study showed differences in their stability of flowering phenology parameters across years or environments, aligning with the results of the studies by Hamze et al. [3], Abou-Saaid et al. [22], and Navas-Lopez et al. [27]. Our study highlights the critical role of G × E interactions in shaping the flowering behavior of the olive tree, with significant variability observed in FBD, FP, and FBP across different environmental conditions. These findings underscore the importance of integrating G × E interactions in variability studies and demonstrate the utility of BLUPs in enhancing the reliability of stability detection [22,31].

**Figure 11:**
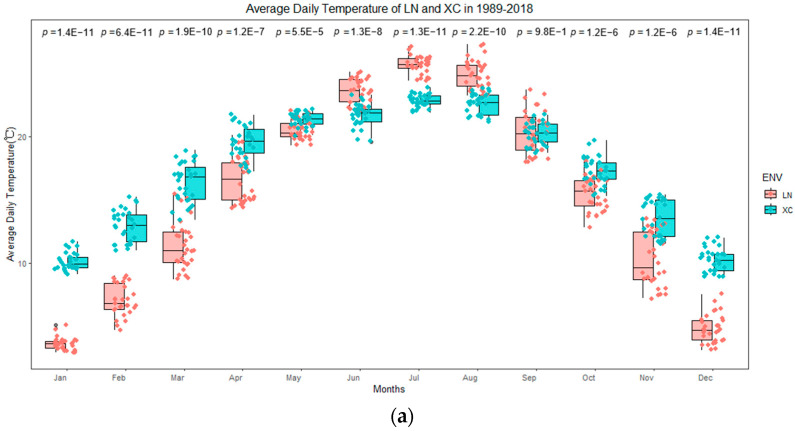

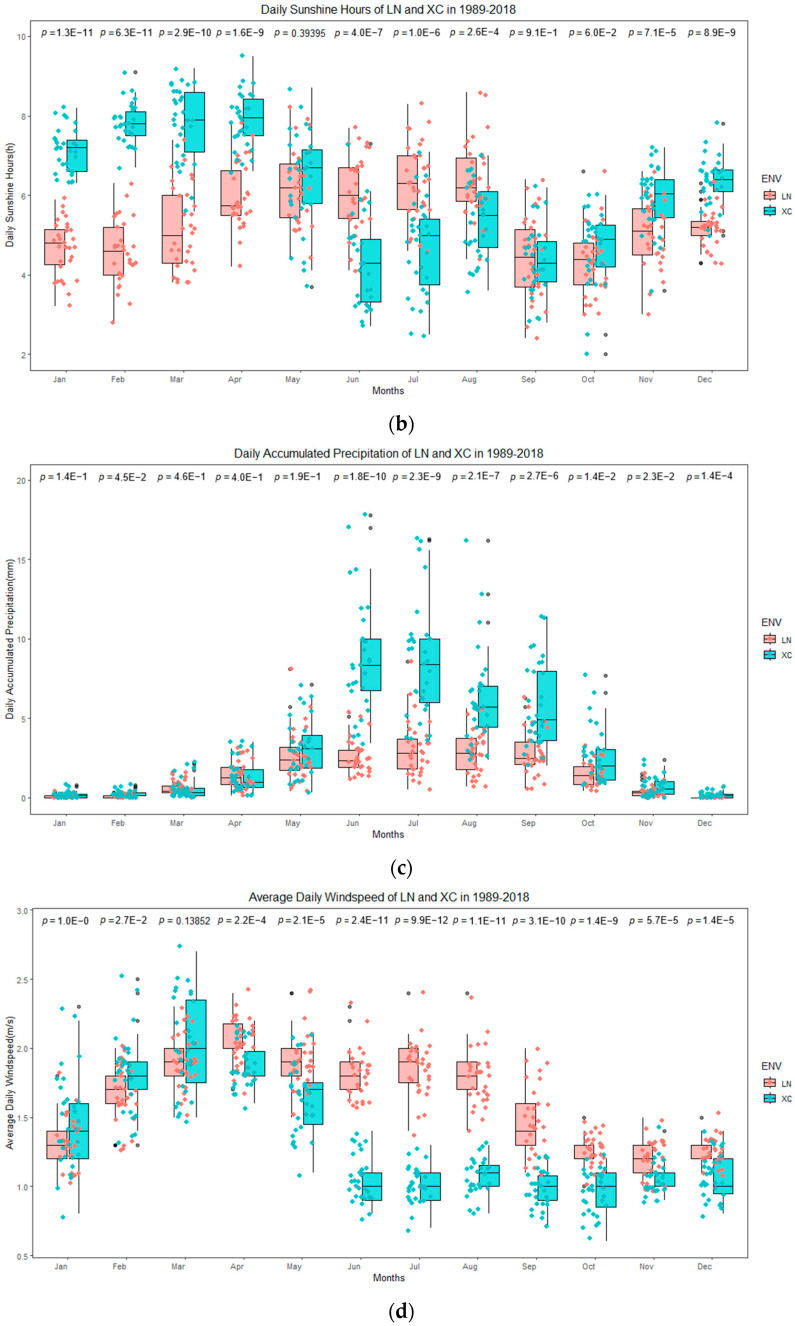
Comparison of the means of climatic data between Longnan (LN) and Xichang (XC) from January to December during the period 1998–2018. (**a**) Average daily temperature; (**b**) daily sunshine hours; (**c**) daily accumulated precipitation; (**d**) average daily windspeed.

The significant GEI effects highlight the challenge of selecting olive genotypes with both high performance and stability. However, innovative approaches and genetic parameter analysis can aid in identifying genotypes that are less affected by environmental variations, particularly temperature variations as experimentally demonstrated by Houssam-eddine et al. [14], Medina-Alonso et al. [12], Hamze et al. [3], Abou-Saaid et al. [22], and Navas-López et al. [38]. The two sites studied, Longnan and Xichang, are characterized by contrasting climatic conditions due to their geographical locations (see Figure 11). These contrasting geographic locations largely explain the different climatic profiles observed across the studied seasons. FBD and FBP traits showed high heritability on genetic mean bases (*h*^2^*_mg_* > 0.5), while FBD and flowering period (FP) had strong GEI correlations (*rge* > 0.952). Heritability varied across traits, with FBP (0.262) and FBD (0.253) showing higher values than FP (0.0589). This is consistent with the finding that relatively higher heritability (*H*^2^ = 0.75) was observed for the full-flowering date (FFD), as reported by Abou-Saaid et al. [22] and Aqbouch et al. [30].

### 3.2. Selection of Genotypes via MPS

The use of MPS index facilitated the identification of olive genotypes that exhibited superior performance and stability in multiple environments. The genotype selection based on MPS highlighted the significance of balancing mean performance with stability, particularly in traits such as FBD, FP, and FBP. For instance, olive genotypes such as CG32, JF4, and ABQ exhibited the lowest BLUP values for FBD, indicating early flowering, while PCH, MS, EZ8, and ABS showed the highest BLUP values for FP, indicating longer flowering periods (Figure 5). These results suggest that targeted selection within specific environments could yield favorable outcomes, especially when considering the significant GEI effects observed in these studies [12,22,27,30].

A WAASB analysis further reinforced the selection of stable genotypes by integrating mean performance and stability [53,56]. The olive genotypes EZ8, ABS, FRT, PCU, LCN, and GRD consistently exhibited low WAASB values across all three flowering traits, indicating their stability under and adaptability to diverse environmental conditions (Figure 7). This finding underscores the importance of using stability indices in breeding programs to identify genotypes that can maintain a consistent performance across various environments. By focusing on the genotypes with both a high mean performance and stability based on the integration of MPS and WAASB analyses, breeding programs can better address the challenges posed by changing environmental conditions and ensure the development of olive cultivars that are well adapted to a range of climates and agronomic practices [5,22,23].

### 3.3. Selection of Genotypes via Multi-Trait Index MTMPS

The MTMPS index offers a comprehensive method for selecting optimal genotypes with high performance and stability across multiple traits. Based on the MTMPS, six superior olive genotypes (EZ8, PCH, KRN, ABQ, JF4, and ABS) were identified, exhibiting the lowest MTMPS values, which indicate their stability and adaptability for the desired traits (Figure 9). The selection differentials (SDs) for FP, FBP, and FBD further demonstrate the potential genetic gains achievable through targeted selection. Higher SD values for FP and FBP suggest significant improvements in these traits (Table 3).

The MTMPS index underscores the value of multi-trait selection in breeding programs, especially considering the complex interplay between flowering traits and environmental factors. It allows the simultaneous evaluation of multiple traits in METs, offering a comprehensive assessment of genotype performance and stability [41,56]. For olive genotypes, flowering phenology is crucial for fruit production and quality [22,23]. The MTMPS index helps breeders identify genotypes with optimal flowering time and duration, ensuring stability across diverse years or environments [42,53]. This is vital due to the impact of climatic factors on flowering dates, affecting yield and oil content [11,14,23,65]. Integrating flowering phenology into the MTMPS framework enables breeders to make better-informed decisions, improving olive cultivar productivity and resilience.

## 4. Materials and Methods

### 4.1. Plant Materials

In this study, sixteen olive cultivars from five different countries (five from Spain, five from Italy, one from Greece, one from France, and four from seedling selection in China) were used in field experiments. The cultivar names, genotype codes, and origin countries are presented in Table 4.

### 4.2. Experimental Sites and Design

Field experiments were conducted at two sites with distinctly different agroclimatic conditions. One site was in Longnan (LN), Gansu Province, in northwestern China, while the other was in Xichang (XC), Sichuan Province, in southwestern China. Analysis of climatic factors, including monthly average temperature, daily precipitation, sunshine hours and wind speed, showed significant differences between the two locations (Figure 11a–d). LN, is located in the Bailongjiang low-mountain valley region at an altitude of 1079 m a.s.l., with the soil type of sandy loam. XC, is located in the Anning River Valley Region at an altitude of 1530 m a.s.l., with the soil type of clay.

Olive cultivars were progressively introduced with the same origin and propagated at the National Base of Improved Olive Varieties in Longnan and Xichang. Trees in both orchards were planted at 4 m × 6 m and maintained with the same olive-growing management aimed at maximizing productivity. Flowering observations were conducted over a period of two years, 2014 and 2015. The field experiments at the two locations were conducted using a random complete block design (RCBD), which included three blocks (replications). Each block represented at least one tree, with a total of at least three trees for each cultivar, aged between 12 and 18 years, which were selected and marked in the orchard at each location. The chosen trees were healthy and have similar canopy size.

### 4.3. Flowering Observations

Flowering observations were conducted to monitor the flowering phenology of the marked shoots and for the sixteen olive genotypes at 2–3-day intervals at the experimental sites in LN from early April and in XC from early March to the end of the flowering period. In the present study, only trees that exhibited significant flowering were included in the analysis. The flowering phenology of the genotypes was evaluated over two consecutive years, 2014 and 2015. The experiments conducted in LN in 2014 and 2015 were designated as LN2014 and LN2015, respectively. Similarly, the experiments conducted in XC in 2014 and 2015 were designated as XC2014 and XC2015, respectively.

The internationally standardized BBCH numerical scale for olive trees [37] was utilized, with observations commencing with the initial appearance of Stage 57 (where the corolla, green in color, becomes longer than the calyx) and concluding when Stage 68 (where the majority of petals have fallen or faded) was the most prevalent. The earliest, most frequent, and latest phenological stages were assessed for each tree on a weekly basis. In accordance with the methodologies proposed by Hamze et al. [3] and Navas-Lopez et al. [27], the aforementioned data were averaged for each tree, block, cultivar, and used to calculate three phenological parameters as follows:

- The length of the flowering period (FP): The number of days from when Stage 61 appeared to have begun to when Stage 68 (where the majority of petals have fallen) appeared to be the most common stage.
- The length of the full-bloom period (FBP): The number of days from when Stage 61 appeared to be the most common stage to when Stage 65 (full bloom, with at least 50% of flowers open) appeared to be the most common stage.
- The full-bloom date (FBD): The average Julian date of the start and end of the FBP, expressed as the DOY (day of the year).

### 4.4. Statistical Analysis

In this study, each combination of year and location was considered to be an environment; therefore, four environments, named Longnan 2014 (LN14), Longnan 2015 (LN15), Xichang 2014 (XC14), and Xichang 2015 (XC15), were considered for the genetic variance and stability analysis of the sixteen olive genotypes.

In accordance with Yue et al. [41], all statistical analyses and data visualizations in this study were performed utilizing the “metan” package v1.18.0 [66] in the software R Studio, R version 4.4.1 [67], with the correlate functions mentioned for each method in the following sections.

#### 4.4.1. Variance Component Analysis and Genetic Parameters

The MET data were analyzed considering the RCBD and using the REML method. An LMM was used to analyze all traits, with random effects used for the genotype, and GEI and fixed effects used for the environment and block within the environment. Variance components and the significance levels of genotypic, environmental, and G × E effects were determined, and the BLUP values for each genotype tested in each individual environment or combined across all environments were calculated with the function gamem_met(). Accordingly, the significance level of the random effects for all the traits studied was tested using the LRT (likelihood ratio test). Genetic parameters, such as heritability, genotypic variance, and the coefficient of variation, were estimated with the argument “genpar” of the function gamem_met().

#### 4.4.2. GGE Biplot Analysis of BLUP

The BLUP values of each genotype in the MET generated for the three flowering parameters were used to perform a GGE biplot analysis with the function gge(). The GGE biplots were used to obtain a graphical representation of the G×E interaction and genotype ranking based on the mean and stability. Graph images were generated based on multi-environment evaluation (which-won-where pattern), genotype evaluation (mean vs. stability), and tested environment ranking (discriminative vs. representative). The genotype ranking was carried out according to the increasing order of each stability parameter. The biplots were constructed on the basis of singular value partitioning  =  2, transformed (transform = 0), environment centered (centering  =  2), and standard deviation standardized (scaling  =  0).

#### 4.4.3. Mean Performance and Stability (MPS) of Single Traits

In order to account for the MPS values of olive genotypes, the WAASBY index was employed, as described by Sampaio Filho et al. [39] and Yue et al. [41]. This index is a superiority index that incorporates the weighting [46]. The WAASBY index was adapted based on the WAAS (weighted average of the absolute scores) from the SVD (singular value decomposition) of the BLUP (best linear unbiased prediction) matrix for the GEI effects generated by the LMM (linear mixed-effects model) and the response variable [46]. The olive genotypes were then ranked using the MPS, which is a superiority index weighted between the mean performance and stability, replacing the WAAS (weighted average of absolute scores) as the stability measure, as at least two IPCAs (interaction principal component axes) are required to calculate the WAASB. The biplots are characterized by the following configuration: the abscissa is represented by the WAASB values on the y-axis, and the ordinate is represented by the response variable on the x-axis. This provides a visual comparison of the value and the stability of the genotypes for a given trait in a two-dimensional plot using all the estimated IPCAs. The function mps() was used to calculate these indices.

#### 4.4.4. Mean Performance and Stability of Multiple Traits (MTMPS)

The MTMPS index was used to evaluate the multi-trait mean performance and stability index of the genotypes [41,44]. The MTMPS index builds on the MPS approach by integrating multiple traits into the stability analysis, thus providing a more holistic evaluation of genotypes than the single-trait MPS approach [46]. This approach is based on an exploratory factor analysis and ideotype design, wherein the factorial scores of each ideotype are formulated based on desirable and undesirable factors. The subsequent estimation of the spatial probability is based on the distance between the genotype and ideotype, thereby allowing for genotype ranking. The results obtained facilitated a single and straightforward process for olive genotype selection. The genotype ranking was carried out based on the Euclidean distance calculated from the score of each genotype to the score of the ideotype. The genotypes exhibiting the lowest MTMPS scores were considered to be the closest to the ideotype and therefore showed higher MPS values across all the traits evaluated [39,41]. The function mtmps() was used to calculate these indices.

#### 4.4.5. Selection Differentials

The selection differential (SD) was calculated for the selected genotypes, assuming a selection intensity of 35%, with a view to determining the selection gain for stability and performance. On the basis of the genotypes selected using the MTMPS index, the calculation of the selection differential (ΔS%) was performed, using the following formula: ΔS% = (XS − X0)/X0 × 100, where XS and X0 represent the values of the selected genotypes and the total mean of all genotypes, respectively.

## 5. Conclusions

This study presents valuable insights into the genotype–environment interaction and genetic parameters influencing olive flowering phenology in non-Mediterranean regions. The observed significant effects of the GEI highlight the challenges in breeding for stable flowering traits, emphasizing the need for targeted selection within specific environments. The use of stability indices, such as the MPS and MTMPS index, offers a robust framework for identifying genotypes with superior performance and stability under diverse environmental conditions.

The findings of this study have important implications for olive breeding programs, particularly in regions with divergent climatic conditions. Future research should focus on expanding the scope of multi-environment trials to include a broader range of genotypes and environmental conditions. Additionally, the integration of advanced statistical tools and multi-trait selection indices could further enhance the efficiency of breeding programs that aim to improve olive flowering phenology and overall productivity.

In conclusion, this study underscores the necessity of considering both genetic and environmental factors in olive breeding initiatives. The identification of stable and high-performing genotypes through targeted selection and multi-trait evaluation offers a promising avenue for enhancing olive cultivation in diverse climatic regions.

## Figures and Tables

**Table 1:** Mean squares obtained from joint ANOVA for FP, FBP, and FBD of 16 olive genotypes in four environments.

Source of Variation	df	FBD	FP	FBP
ENV	3	2.45 × 10^4^ ***	969.924 ***	76.102 ***
REP(/ENV)	8	3.80 × 10^−1^ ***	0.193	0.922 ***
GEN	15	3.72 × 10^1^ ***	13.065 ***	6.175 ***
GEN:ENV (GEI)	45	1.57 × 10^1^ ***	10.379 ***	2.262 ***
Residuals	120	8.02 × 10^−2^	0.170	0.244
CV (%)		0.241	3.11	11.70

Note: ***, *p*  <  0.001; ENV, environment; REP, replicate; GEN, genotype; GEI, genotype × environment interaction; df, degrees of freedom; FBD, full-bloom time by Julian date; FP, length of flowering period; FBP, length of full-bloom period.

**Table 2:** Genetic parameter estimates for flowering traits of 16 olive genotypes evaluated in four environments. Note: PV, phenotypic variance; *h*^2^*_mg_*, heritability of genotypic mean; GEIr^2^, GEI coefficient of determination; AS, accuracy of selection; *rge*, correlation between genotypic value across environments; *CVg*, genotypic coefficient of variation (%); *CVr*, residual coefficient of variation (%); *CV ratio*, ratio between the coefficients of genotypic and residual variations (%); FP, length of flowering period; FBP, length of full-bloom period; FBD, full-bloom time (by Julian date).

Parameters	FBD	FP	FBP
PV	7.08	3.80	1.24
heritability	0.253	0.0589	0.262
GEIr^2^	0.736	0.896	0.541
$h_{mg}^{2}$	0.578	0.206	0.634
AS	0.760	0.453	0.796
*rge*	0.985	0.952	0.734
*CVg*	1.14	3.56	13.5
*CVr*	0.241	3.11	11.7
*CV ratio*	4.72	1.15	1.16

**Table 3:** Selection differentials (SDs) of the mean performance and the WAASBY index for the three traits.

	Mean Performance							WAASBY				
Trait	Factor	Xo	Xs	SD	SD%	Sense	Goal	Factor	Xo	Xs	SD	SD%
FP	FA1	13.28	13.69	0.413	3.11	Increase	100	FA1	47.2	60.4	13.1	27.8
FBP	FA1	4.22	4.53	0.304	7.19	Increase	100	FA1	55.6	66.2	10.5	18.9
FBD	FA2	117.7	117.4	−0.299	−0.25	Decrease	100	FA2	58.3	66.1	7.80	13.4

Note: SD, selection differential; Xo, population mean; Xs, mean of selected genotypes; FP, length of flowering period; FBP, length of flowering-bloom period; FBD, full-bloom time by Julian date.

**Table 4:** Origin country and genotype code of the studied olive cultivars.

Cultivar Name	Origin Country	Genotype Code	Cultivar Name	Origin Country	Genotype Code
Arbequina	Spain	ABQ	Leccino	Italy	LCN
Arbosana	Spain	ABS	Pendolino	Italy	PDL
Gordal	Spain	GRD	Koroneiki	Greece	KRN
Manzanilla de Sevilla	Spain	MS	Picholine	France	PCH
Picual	Spain	PCU	Chenggu32	Seedling selection	CG32
Ascolano Tenera	Italy	AST	Ezhi8	Seedling selection	EZ8
Coratina	Italy	CRT	Jiufeng4	Seedling selection	JF4
Frantoio	Italy	FRT	Zhongshan24	Seedling selection	ZS24

## Data Availability

The data underlying this article are available in the article.

## Abbreviations

The following abbreviations are used in this manuscript:

FBD	full-bloom date
FP	flowering-period length
FBP	full-bloom period length
BLUP	best linear unbiased prediction
MPS	mean performance and stability
MTMPS	multi-trait mean performance and stability
METs	multi-environment trials
REML	restricted maximum likelihood
LMM	linear mixed model
LRT	likelihood ratio test
WAAS	weighted average of the absolute scores
SVD	singular value decomposition
IPCAs	interaction principal component axes
SD	selection differential
GEI	genotype–environment interaction
ANOVA	analysis of variance
GGE	genotype + genotype by environment interaction
